# Obstacles in Haemocompatibility Testing

**DOI:** 10.1155/2013/392584

**Published:** 2013-05-07

**Authors:** W. van Oeveren

**Affiliations:** HaemoScan and Department of Cardiothoracic Surgery, UMCG Groningen, The Netherlands

## Abstract

ISO 10993-4 is an international standard describing the methods of testing of medical devices for interactions with blood for regulatory purpose. The complexity of blood responses to biomaterial surfaces and the variability of blood functions in different individuals and species pose difficulties in standardisation. Moreover, *in vivo* or *in vitro* testing, as well as the clinical relevance of certain findings, is still matter of debate. This review deals with the major remaining problems, including a brief explanation of surface interactions with blood, the current ISO 10993 requirements for testing, and the role of *in vitro* test models. The literature is reviewed on anticoagulation, shear rate, blood-air interfaces, incubation time, and the importance of evaluation of the surface area after blood contact. Two test categories deserve further attention: complement and platelet function, including the effects on platelets from adhesion proteins, venipuncture, and animal derived- blood. The material properties, hydrophilicity, and roughness, as well as reference materials, are discussed. Finally this review calls for completing the acceptance criteria in the ISO standard based on a panel of test results.

## 1. Introduction

Application of biomaterials in direct blood contact results in activation of the blood coagulation system and in an inflammatory reaction. These responses of blood are due to the natural response of the host defence mechanism against foreign surfaces. Inadequate control by natural inhibitors results in pathological processes, such as microthrombi generation or thrombosis, bleeding complications, haemodynamic instability, fever, edema, and organ damage. These adverse events become manifest during prolonged and intensive foreign material contact with vascular implants and extracorporeal blood circulation [1–7]. Despite their small surface area, coronary stents impose an ongoing risk for thrombosis by 1% per year [8]. A major difference between blood contacting and tissue implants is that blood might continue to be activated, despite a covering layer of proteins and cells, whereas implants in tissue become encapsulated and thus neutralised within weeks. It is hard to speculate on a direct relation with haemocompatibility, but consensus exists about the important role of poor haemocompatibility in direct and sustained adverse reactions. This review will deal with some of the remaining important practical aspects and problems of haemocompatibility testing. Adequate positive reference materials are warranted and acceptance criteria for all aspects of haemocompatibility should be in place.

Determination of haemocompatibility can be studied accurately by adequate *in vitro* models, since disturbing factors such as surgery, tissue effects, and flow obstruction can be avoided, while blood contact is more intense and activation products are not cleared. *In vitro* models are suitable to be used with fresh human blood, which is relevant since responses of haemostatic and inflammatory systems are essentially different between human and animal blood.

## 2. Surface Interactions of Blood

After contact of blood with a material various proteins will be deposited within split seconds. The main proteins adhered to a surface are albumin, fibrinogen, and immunoglobulin, based on their high concentrations in blood. After the initial adhesion a continuous exchange with free proteins takes place, that reaches equilibrium after approximately two hours. This results in binding of higher molecular weight proteins. The relatively medium molecular weight protein albumin will be exchanged in part for larger proteins. Next to nonspecific protein deposition, some components of the contact system react specifically with negatively charged surfaces. As soon as the blood comes in contact with a negatively charged surface, Factor XIIa fragments are formed. These fragments then initiate the entire contact system. ß-Factor XIIa converts prekallikrein into its active form, kallikrein, which generates the vasodilator bradykinin [9]. The deposition and conformation of some plasma proteins on the artificial surface, such as Factor XII, fibrinogen, and vitronectin are a significant criterion for further thrombogenicity [10]. After deposition to some surfaces fibrinogen leads to a strong adhesion of platelets through platelet glycoprotein receptors (GpIIbllla), followed by platelet aggregation, and release of procoagulant contents from platelets. Additionally, contact activation induces activation of the coagulation cascade. The adhered platelets release adenosine diphosphate and activate the arachidonic acid synthesis pathway to produce thromboxane A2 [11]. Thromboxane A2 is a potent chemoattractant and smooth muscle cell mitogen and leads to further platelet recruitment [12]. Upon activation and during apoptosis platelets and other cells bud off small parts of their plasma membrane, called microparticles (MP). 

These aforementioned processes result in thrombosis, which is the deposition of various blood elements onto a surface. Thrombosis can be formed in blood vessels, but also on artificial materials placed in the blood circulation or outside the body in extracorporeal circuits such as dialysis and a heart-lung machine. Since a thrombus is formed by the various blood elements, it can also be formed in circulating blood separate from the organism in *in vitro* models. 

The inflammatory reaction is initiated by complement activation. C3b, which is present in small amounts in blood, forms a C3 convertase (C3 cleaving enzyme) when not immediately degraded by complement inhibitors. Since foreign surfaces lack complement inhibiting capacity, the complement convertases will amplify the complement reaction, resulting in more C3b generation and its deposition onto the surface. Simultaneously, the smaller C3a fragment is released in plasma and this fragment is often used as a marker of complement activation. Thus, an exponential activation of the complement system takes place after recruitment of the other complement factors. After C3 convertase C5 convertases will be formed on a material surface, with cleavage capacity for C5 into C5a and C5b.

Complement has lytic effects on target cells by its end stage components C5b-9 (terminal complement complex) and therefore may become harmful for the patient in contact with an activating device. In addition, much of the deleterious effects of complement activation is related to the recruitment and activation of leucocytes, such as granulocytes and monocytes. Granulocytes show an upregulation of the adhesion molecules CD 11 and CD 18 with increased adhesion to the surface, release of elastase, and superoxide generation, that is, further propagation of the inflammatory response [13, 14].

## 3. ISO 10993 Requirements for Testing of Medical Devices: Is an *In Vitro* Model Sufficient to Replace Animal Models?

In December 2002 the revised ISO 10993-Part 4 standard (*Biological evaluation of medical devices*—*Selection of tests for interactions with blood*) was published [15]. Its revision will be finished approximately 12 years later and is currently undergoing major revisions due to new insight into the importance of complement activation and positioning of *in vitro* models. The standard is applicable to external communicating devices, either with an indirect blood path (e.g., blood collection devices, storage systems) or in direct contact with circulating blood (e.g., catheters, extracorporeal circulation systems), and implant devices (stents, heart valves, grafts). Testing should be performed for five categories, based on primary processes: thrombosis, coagulation, platelets, haematology, and complement. In this system all relevant aspects of blood activation are taken into consideration, but, and this is most important, testing should simulate clinical conditions as much as possible. It is questioned whether *in vitro* models could also be used to similate clinical conditions and to generate thrombosis. Since thrombosis is the formation, presence, or development of a thrombus [16], it can be argued that thrombosis is an *in vivo* as well as an *in vitro* phenomenon [17, 18].

 One important and extremely relevant aspect of testing of medical devices is the condition of blood exposure to the device. Often, blood with clinically inapplicable anticoagulants and under static conditions was incubated with the test device [19–21]. Currently anticoagulation and flow conditions must be as similar as possible to the clinical application to achieve relevant test results. Thus, most devices must be tested with heparinised blood under circulating conditions. For some devices, such as stents and catheters this implies high flow through or around the device to obtain relevant shear stress conditions. The major differences observed between cell interaction under static and flow conditions have made clear that whole-blood flow models are required for testing haemocompatibility inasmuch as the test device will be used clinically in the blood circulation. Flow models for testing may consist of animal models or *in vitro* test systems. Animal models have the disadvantage of being expensive, time consuming, and insensitive due to overwhelming short-term effects of tissue damage. Moreover, it has been shown that the composition of blood differs considerably between various species, which leads to over- or under estimation of human blood reactions to biomaterials [22, 23]. The use of human blood is therefore more relevant to the interpretation of results and offers a more detailed array of test methods, since most available methods are based on human blood components. The use of human blood requires a proper *in vitro* circulation model.

An *in vitro* blood circulation model is very attractive to test haemocompatibility of materials or small devices. Small volumes allow duplicate or triplicate testing of controls and reference materials together with test objects with the same batch of blood and at the same time. It allows the use of human blood, while flow, temperature and anticoagulation can be standardised. An *in vitro* model on the other hand may be regarded a worst case scenario, since activation products accumulate without clearance by kidneys or liver or other organs, whereas activation-inhibiting functions of endothelial cells are absent. Enforced by these circumstances the reactions of blood with biomaterial are *in vitro* much faster than *in vivo*. A well-designed future study could provide insight into the optimum time of *in vitro* blood contact. 

### 3.1. Anticoagulation

Anticoagulation during clinical application of medical devices consists of unfractionated heparin. This inhibits Factor Xa and thrombin by enhancing the natural inhibitor antithrombin III. Since a commonly used clinical concentration of 1,5 IU/mL heparin is sufficient to prevent clotting *in vitro*, it is very sensible to use this concentration during testing of medical devices. Although heparin has some inhibiting effects on the complement system [24], this effect is only measurable at concentrations 1000 times higher than clinically used. Inhibitor of complement heparin can therefore be neglected as compared to citrate or EDTA, which bind calcium, a cofactor for the clotting as well as the complement system. Since heparin does not completely prevent activation of the coagulation cascade, a fair amount of thrombin-antithrombin III is formed during blood circulation. When strong activators of the coagulation system are used or when platelets are activated to a high extent heparinised blood may totally clot. However, during 1–4 hours of *in vitro* circulation this is not very common. Platelet activation contributes to clotting by not only exposure of activated membranes and release of clotting factors but also by release of platelet factor 4, which is neutralising heparin. Some *in vitro* models require other methods of anticoagulation or no anticoagulants at all [25]. Platelet studies require sometimes PPACK26, to inhibit only formed thrombin, whereas hirudin shows less aspecific inhibition than heparin [26]. In contrast, higher concentrations of heparin may be required to control coagulation sufficiently [27]. Anticoagulation with citrate showed more platelet adhesion than heparinized platelet-rich plasma [18].

### 3.2. Shear Rate

A key determinant of blood activation and adhesion of cells is wall shear stress, the force exerted by the flow per surface area. In a cylindrical tube this property is easily calculated from *τ* = 32*Qη*/(*πD* ^3^) where *τ* is the shear stress, *Q* is the flow rate, *η* is the viscosity, and *D* is the tube diameter [22]. In configurations that differ from the cylindrical tube, for example, just after a bifurcation, wall shear stress has much larger values locally, than at the opposite site [28, 29]. Growth of intimal thickness is often observed at locations with low shear stress [30]. Wall shear stress in the normal circulation is rather constant when the equation is applied to blood vessels of various sizes [31]. Values are found in the range 10–20 dynes/cm^2^ (1-2 Pa). Furthermore, blood vessels adapt their diameter as much as possible towards a constant value for shear stress [32].

Different reactions occur in blood under low or high wall shear rate. If certain blood reactions are ignored, or if the material is not assessed under appropriate flow conditions, the material will not be well characterized as to its interaction with blood. For example, when blood is in contact with biomaterial surfaces, fluid mechanics, but especially shear stress, have a strong influence on the damage of erythrocytes and platelets. Red cell damage may occur at high shear stress [33, 34].

Platelets are more easily damaged by shear stress [33, 35]. Platelet damage is not only influenced by the maximum shear, but also by the duration of the shear force. Only for very short exposure times platelets are able to withstand higher shear stress than erythrocytes [35].

From a fluid mechanical point of view, differences in flow situations may therefore lead to different problems with blood. Artificial heart valves may cause problems for erythrocytes due to short duration high shear [36], whereas stents in the coronary arteries induce intimal growth at locations of relatively low shear [37], which may be caused by platelet activation in high shear. Neointima formation in stents has been shown to be related to wall shear stress as well [37]. In tubing used during dialysis, the high shear rate at the needle may lead to problems for erythrocytes [38], but it should not be disregarded that the wall shear stress of the tubing is the most critical issue for platelet activation.

Heart valves, extracorporeal systems, and vascular grafts or stents all induce relatively high shear forces that may result in platelet activation. Shear stress is a natural activator of platelets. The shear-induced pathway appears to be one of the major pathways of platelet-induced haemostasis and thrombosis [39]. The sequence of the shear-induced pathway is the binding of von Willebrand Factor (vWF) to the platelet glycoprotein Ib (GpIb) receptor, the expression of activated GpIIbIIIa receptors and release of platelet vWF. VWF multimers are large molecules, which bind with high affinity to artificial surfaces. Finally, GpIIbIIIa binds to vWF, leading to irreversible adhesion. High shear stress of 120 dynes/cm^2^ induces immediate expression of GpIIbIIIa receptors, and release platelet vWF multimers [40]. However, in the presence of platelet activators, such as epinephrine and ADP, shear stresses of 60 dynes/cm^2^ may synergistically result in platelet aggregation.

 Fibrinogen appears to mediate platelet aggregation efficiently at low shear rates, but not at high shear rates [41]. Moreover, resting platelets do not adhere efficiently to fibrinogen-coated surfaces; activation by ADP is required [42]. It has been demonstrated that the conformation of fibrinogen and not the concentration of fibrinogen on a material surface induces platelet adhesion and activation [43]. 

### 3.3. Blood-Air Interface

Clinical use of extracorporeal circuits in open-heart surgery has provided evidence that blood-gas interfaces activate different blood cascades of hemostasis [44, 45], inflammation [45, 46], and tissue damage [47]. Also in an *in vitro* model complement activation by blood-gas interface has been observed [48]. Despite this, the classical Chandler loop [49], *in vitro* test model, or modifications thereof [50] are still widely used to test haemocompatibility. The Chandler loop consists of a closed tubing partly filled with air, which circulates the device constantly through an air-liquid interface. Not only are blood cascades activated, but also this method may induce artifacts due to the major forces applied on blood elements and to protein denaturation at the air-liquid interface [51–54]. Noteworthy is the defouling and cleaning effect of air bubbles [55–57], potentially washing off adhered blood cells. Thus, instead of the Chandler a small roller pump closed-loop system was used in the past. This model appeared effective for short-term circulation [58–61]. However, blood damage induced by the pump limited the exposure of the test object to circulating blood. Since metal stents are commonly of a thrombotic nature, experiments for 15 minutes yielded sufficient information to compare stents, but a less traumatic circulation system is required for testing of low thrombotic materials. Since improvement of the model by minimising blood damage may increase sensitivity and permits prolonged blood exposure, a simple mechanical device without air and without a pump to reduce blood damage and activation by the device is required [62]. Moreover, pulsatile flow at a frequency similar to the arterial circulation can be generated by a new version of such device [63], which appeared to reduce intrinsic blood damage. Pulsatile flow generates 20% more platelet adhesion on collagen than only high flow [64] and more than double compared to low flow, indicating the importance of simulating the corresponding clinical conditions [65]. 

A limitation of *in vitro* models is mainly represented by the absence of an endothelial layer in the circulating system. Throughout the release of (anti) thrombotic components and the expression of adhesion molecules, endothelium has a major role in mediating the interplay between the injured vessel wall and blood cells after biomaterial implants [66, 67] and lack of this character can somehow alter the likelihood of the experimental representation. Nevertheless, all the other elements depicting the blood-material phase boundary scene are present in the *in vitro* model.

### 3.4. Incubation Time *In Vitro*

The effect of blood-material interaction in an *in vitro* model is accumulated due to the continued contact, absence of component removal by organ function, and high surface/volume ratio. Thus it may be imperative to limit the blood contact time in order to avoid plateauing of effects due to exhaustion of activation products. In particular platelets [68] and the complement system are rapidly activated and also rapidly exhausted as can be seen from affected platelet function after circulation in an *in vitro* model [69] and from optimum activation times of the complement system after 15–20 minutes [70]. Platelet adhesion is a very rapid phenomenon, which is taking place in seconds to minutes [17]. Results of time-course measurements of the variables may importantly contribute to establishing a proper *in vitro* test protocol. Experiments with extended circulation in a Chandler model showed a continued increase of complement activation [71]; however most of the TCC was generated within 75 minutes, indicating that the first contact caused a major effect on the complement system. Since prolonged circulation does not further differentiate between materials, it may be caused by material-independent effects, like protein denaturation or aggregation. 

### 3.5. The Relevance of Surface Evaluation after Blood Contact

During direct contact of blood with biomaterials in an *in vitro* test system three sources of information can be collected. The first is deposition of proteins or formed elements on the material surface. The second is the changes of platelets, leucocytes, or erythrocytes in circulation, and the third is the activation products formed or released in plasma.

Examples of surface deposition are the complement convertases or TCC [72–75], fibrinogen, immunoglobulins, albumin, platelets, and leucocytes or their receptors. Deposition of these components represents the most direct effects of the material and the deposition is the trigger for further activation of blood. In addition, by separation of the materials after blood contact, the surface deposition provides results almost independent of the *in vitro* or *in vivo* confounding factors. In this regard, high sensitivity and specificity were obtained by measurement of surface bound complement convertase and platelets and by scanning electron microscopy visualisation of thrombus formation.

Secondly, leucocytes and in particular platelets that have been in contact with a biomaterial will be activated or damaged. Measurement of cell count, receptor expression, or functional changes might refer to the effects of the biomaterial, although rheological effects cannot be excluded.

Finally plasma collected after *in vitro* circulation can be used for determination of activation products. This is the easiest procedure, but unfortunately also the most indirect method and prone to artifacts. Complement activation, thrombin formation, and platelet release products are rapidly generated and accumulate whenever blood is kept or circulated through an artificial device without inhibitory agents, such as EDTA. The most commonly tested complement components are C3a, C5a, and C5b-9 (terminal complement complex). In all publications plasma concentrations of these markers are increased 10 to 50 times of normal even in the presence of low activating materials [76–79]. It is evident that inhibition prior to circulation is not an option in order to exert an experiment which resembles *in vivo* blood contact. The high concentrations of C3a, C5b-9, thrombin-antithrombin III, and beta-thromboglobulin without marked differences between low and high reference materials are indicative of the low selectivity of plasma activation markers [80].

## 4. Complement

Complement activation induced by biomaterial has become an important variable of haemocompatibility. It would be of value to be able to use the extent of complement activation in a classification and to relate the classification of high activation material to clinical disorders.

From the literature it has become clear that *in vivo* much lower plasma concentrations have been detected than after *in vitro* blood contact, probably due to clearance *in vivo* in contrast to accumulation in a small volume of blood *in vitro*.

As a rule of thumb ten times increased concentrations of C3a, C5a, and C5b-9 are found after surgery or infection, whereas 100 times increased concentrations were found after *in vitro* exposure of blood at 37°C.

### 4.1. Clinical Effects of Complement Activation and Acceptance Criteria

Uncontrolled activation of the complement system during sepsis and systemic inflammatory response syndrome (SIRS) with excessive generation of complement activation products contributes to an ensuing dysfunction of various organ systems [81]. To characterize multiorgan failure the lungs, cardiovascular system, kidneys, liver, coagulation system, and central nervous system are regarded the most sensitive [82–84]. There is evidence that all three complement activation pathways are activated in SIRS and sepsis [85–87], whereas the alternative pathway is predominantly activated by biomaterials and the classical pathway by surgery. 

C1q, C3a, and C5a contribute to intracranial inflammation by induction of blood brain barrier damage and increase in vascular permeability [88, 89]. 

The lungs have been shown to be affected by C3a and C5a and by increase of their receptors [90]. C4a, C5a, and C5b-9 complexes are related to adult respiratory distress syndrome pathophysiology [91–95].

C5a causes the local release of cardiosuppressive cytokines and chemokines in cardiomyocytes eventually leading to cardiac dysfunction [96]. But it is also conceivable that complement anaphylatoxins contribute to induce “hibernation” in cardiomyocytes as it occurs in the response of the myocardium to ischemia [97]. 

The liver may become increased susceptible for complement-mediated cytotoxicity of hepatocytes during sepsis. 

Kidney injury marker KIM-1 expression was upregulated by the stimulation of C3a, C5a, or C3/C5a. C3 deposition, and no evidence for C4 deposition, along tubules could be found in acute tubular necrosis after renal ischemia/reperfusion injury, indicating that the alternative pathway is the predominant complement activation pathway for the development of acute tubular necrosis [98]. C3a and C5a have vascular effects that contribute to changes in renal hemodynamics in acute renal failure [99, 100]. This change was evident after 5 min and maximal after 20 min. This occurred at C3a concentrations >100 ng/mL. 

Disseminated intravascular coagulation (DIC) represents a frequent complication after trauma, systemic inflammation, and sepsis [101, 102]. After the initial phase of hypercoagulability with intra- and extravascular fibrin clots, consumption of coagulation factors and dysfunction of thrombocytes can lead to hemorrhagic diasthesis and diffuse bleeding [101–103]. Intravascular fibrin clots are finally responsible for impaired microcirculation and hypoxic cellular damage [103]. Trauma, thermal injury, and infection predispose to thrombosis and the development of DIC and trigger the inflammatory response including complement activation, which, in turn, can trigger coagulation and vice versa [102, 104]. Thrombin is capable of cleaving C5, resulting in the generation of C5a. 

C5a interacting with its receptors (C5aR, C5L2) triggers a cascade of destructive outcomes including inhibition of innate immune signaling pathways, leading to impaired phagocyte function (e.g., phagocytosis, chemotaxis, and the oxidative burst) [105].

These effects of complement products and in particular C3a, C5a, and C5b-9 on organs show that concentrations of the anaphylatoxins around 100 ng/mL can cause direct damage to various organ systems. C5b-9 may be devastating at lower concentrations due to its direct lytic effects. *In vitro* experiments demonstrate complement activation by exposure of blood to biomaterial of 10–500 ng/mL [106]. This shows that potentially biomaterial and in particular large blood contacting devices, such as heart-lung machines and hemodialysers in the systemic circulation, can cause complement activation at pathologic concentrations. Locally, smaller biomaterial surface may induce high concentrations of complement activation products. If a standardised surface-area versus blood volume ration is respected, it seems possible to define a certain threshold above which complement activation can be considered potentially harmful for patients. Such threshold is around 10 times baseline values. The interpretation of testing could be improved when such criteria would be implemented.

## 5. Platelet Adhesion and Function

The contact of blood with a body foreign surface initiates a cascade of processes: (1) protein adsorption at the surface, (2) adhesion of platelets to the body foreign surface via adherent proteins, (3) activation of further neighbored platelets, and (4) the stabilization of the thrombi by fibrin in a local network structure [107]. In flowing blood platelets are of much more importance than clotting in inducing a thrombus, because of their immediate reaction. Therefore platelet function testing should get more attention in haemocompatibility studies than in the past. Since the four processes described here regarding platelet interaction are not measured with extracts of a biomaterial, it is evident that blood-material contact is required to evaluate the response of platelets.

### 5.1. Protein Adsorption

Immediately after the contact of the subendothelium or body foreign surfaces with blood, adsorption of plasma proteins occurs [108–110]. The adsorption of plasma proteins determines the subsequent interaction of blood cells with the body foreign surface. The time necessary to adhere to body-foreign surfaces depends on the protein, the chemistry, and surface characteristics of the material itself. The most important proteins in this process are fibrinogen, vitronectin, fibronectin, immunoglobulins, vWF, HMW-kininogen, prekallikrein, Factor XI, and Factor XII. But one should be aware that there are more than 5000 platelet proteins [111], of which more than 300 are released during activation [112]. Fibrinogen (Fg) has been identified as one of the most important types of adsorbed proteins that induces a platelet adhesion response, especially at medium shear rates [113]. It has been shown that the platelet adhesion response did not correlate to the amount of the fibrinogen adsorbed, but to the adsorption-induced conformational changes of the fibrinogen and albumin [114]. A similar conformational change of the natively globular von Willebrand molecule during adhesion was shown as well [115]. This also explains why under physiological conditions no interaction with nonactivated platelets occurs. 

### 5.2. Platelet Activation and Adhesion

Normal, nonactivated platelets are small nuclear cell fragments of 1-2 um. During their life time, of 10 days most platelets never adhere. Only when the endothelial cell layer is damaged or in case of biomaterial exposure, the adhesive potential of platelets becomes evident. The adhesion of platelets to the subendothelial matrix or body foreign surfaces is the initial step in primary hemostasis. Platelets interact with adherent proteins via more or less specific adhesive glycoproteins (GP). Binding of biochemical agonists to their receptors, receptor cross-linking, or changes in the plasma membrane induce a complex cascade of signals, transduced from the membrane into the cytoplasm resulting in platelet activation [116]. After surface adhesion, spreading, activation, secretion, and lastly aggregation with further platelets may take place [117]. Platelet activation is a very fast process of *∼*180 ms [118] so that platelets passing by can be activated and then adhere. Activated platelets show a change in the assembly of cytoskeleton proteins, resulting in a shape change with extensive formation of pseudopodia originating from the plasma membrane [119]. Thereafter, a spreading of platelets occurs—which takes 1 to 3 minutes requiring nonactivated functional platelets with normal energy metabolism [120]—accompanied by the release of substances, which act as vasoconstrictors (thromboxane, platelet-derived growth factor (PDGF), serotonin), promote adhesion of further platelets in the surrounding plasma (fibronectin, von Willebrand Factor), and mediate the aggregation of neighboured platelets. Platelets attach to body foreign surfaces by a complex series of events. They adhere to surfaces via different receptors (glycoproteins (GP)) depending on the nature of the material, the structure, and charge of the surface and the prevailing rheological conditions [121–123]. Blood flows with a greater velocity in the center of the vessels than near the wall, thereby generating shear forces between adjacent layers of fluid that become maximal near the vessel wall. In such regions of high shear stress, platelets can also become activated only by shear forces (greater than 500 dynes/cm^2^ [124]). Since they circulate near the vessel wall [125]—in the so-called cell free plasma layer—such activated platelets are able to adhere to body foreign surfaces or injured or denuded vessels walls.

Various approaches aimed at developing more blood compatible polymeric materials which are currently being investigated in research laboratories worldwide. They can be generally categorized into methods based on (1) mimicking nonthrombogenic endothelial cells (EC) [126–130] which line the inner walls of all healthy blood vessels and (2) use of chemical surface moieties that suppress blood-material interactions (e.g., polymeric surfaces that exhibit decreased protein and cell adhesion). Adsorption of proteins (especially fibrinogen and von Willebrand's factor) is the first step in the overall process by which blood contacting materials can activate platelets, which leads eventually to thrombus formation on or near the surface of the implanted device. Albumin, with around 40 mg/mL the main protein constituent of plasma [131], is considered to be nonadhesive to platelets, as it lacks any known motifs (amino acid sequences) for binding platelet receptors [132]. It has been considered to be unable to support platelet adhesion and hence is widely used for blocking nonspecific platelet-surface interactions in platelet adhesion studies [132] as well as for a hemocompatible coating for biomaterial surfaces [133–135]. Since it is difficult to prevent replacement of albumin by other proteins, approaches that mimic the nonthrombogenic EC appear to be promising. Molecules contributing to the nonthrombogenic/anti-platelet properties of the EC include nitric oxide (NO) (generated from l-arginine by nitric oxide synthase (NOS) within the EC), thrombomodulin, prostacyclin, and heparins [136, 137]. Hence, if polymers can be prepared that can release and/or possess immobilized forms of some of these species, the surfaces of such materials are likely to be more thromboresistant [138].

### 5.3. Platelet Activity after Venipuncture

It is extremely important to use freshly prepared platelets since platelets age rapidly outside the vascular system. As early as one hour after collecting a blood sample, the reactivity of platelets to pharmacological agonists decreased significantly [139–141]. 

In healthy donors, on average about 6% of the platelets are activated [139] while in patients with atherosclerosis up to 30%–40% of platelets are activated [142]. Also, in early stages of an infectious disease, the number of activated platelets might be increased before any clinical hint is observable. It is also of importance that subjects included in a haemocompatibility study should not ingest a fatty meal some hours before the study, because it might also lead to an elevated percentage of activated platelets [143], while sea food, chocolate, honey, a lot of different vegetables, strawberries, or coffee may lead to a decrease of hyperaggregable platelets [140, 142–148]. Also, errors possibly occurring during blood sampling due to a too high shear stress in the syringe cannula should be avoided. Therefore, the inner diameter of the cannula (needle of the syringe) should not be less than 1.1 mm (19F) and the anticoagulant should be mixed with the blood while it is collected.

### 5.4. Human or Animal Platelets?

Human platelets were compared with canine and porcine platelets with regard to membrane lipid composition. Significant differences in several lipid concentrations were observed, some of which involved major lipid constituents [149, 150]. In addition the morphology appeared to be markedly different between bovine and human platelets [151]. This likely contributes to variations in platelet responses to stimulation between species.

The differences seem to be expressed in functionality, such as adhesion to foreign surfaces. In various studies major differences were observed between humans compared to rabbit, canine, calf, sheep, and porcine platelets [152], with regard to aggregation [153] and response to agonists and inhibitors [154]. While pigs are often used today as an experimental animal for cardiovascular studies [155], the interaction of porcine platelets with surfaces is essentially different due to the minor role of the GpIb-von Willebrand interaction and phosphorylation profile [156]. These major differences between human and porcine platelets are also shown by modern hemostatic techniques, such as thromboelastography [31].

All authors who compared human with animal platelets concluded that results from animal studies cannot simply be applied to outcome after clinical use. As a result it is advisable to use human blood for *in vitro* systems of hemocompatibility testing.

## 6. Biomaterial Surface Characteristics in relation to Haemocompatibility and Clinical Applications

Adhesion and activation of platelets to biomaterials surfaces are an important step in thrombosis and are governed, in part, by surface energy and wettability of the biomaterial surface [157]. Prior to adhesion of platelets, plasma proteins like fibrinogen and fibronectin adsorb [158], and the composition of the adsorbed plasma proteins relates to the wettability of the biomaterial surface [159, 160]. Adhesion can be controlled by adjusting the surface properties—especially surface energy—of the material involved. Long-term implantation of totally artificial hearts is one of the most compelling proofs of the bioengineering utility of surface energy modification to minimise biological adhesion. These pumps, and the related intra-aortic balloons and left ventricular assist devices, do not accumulate blood clots or thrombotic masses during their contact with blood. Negatively charged surfaces do repel blood cells which are also negatively charged. By their negative charge amorphous hydrogenated carbon or silicon-carbide film provide a thromboresistant surface [161, 162]. 

### 6.1. Hydrophilic or Hydrophobic Properties

The wettability of biomaterials relates to the extent of hydrophobicity and hydrophilicity. An increased wettability on a polymer gradient was related to an increased amount of oxygen incorporated in the material surface and appeared to correlate with increased protein adhesion, activation of the coagulation system, and increased platelet adhesion [162]. *In vitro*, shear force induced platelet activation and adhesion to collagen occurs within 2 s, with half of the number of reacting platelets adhering within 240 ms [163]. This is important for efficient haemostasis under flow conditions and in the contact of blood with medical devices. Generally, more platelets adhere to the hydrophilic than to the hydrophobic surfaces, while flow promotes platelet adhesion evidently through increased convective mass transport [164]. Moreover, attachment is known to be stimulated by shear stress, which causes haemostasis under arterial flow conditions [165, 166].

A moderate flow and shear stress (0.8 N/m^2^) generated most platelet adhesion on a hydrophobic surface. However, when the flow was further increased to simulate the conditions of coronary arteries at 3.2 N/m^2^, platelet numbers at hydrophilic surfaces were significantly reduced as compared with hydrophobic surfaces [167]. These results strongly suggest detachment of platelets from hydrophilic surfaces, which can be explained by the small contact area of platelets [168]. Furthermore, the platelets attached to hydrophilic surfaces remain spherical and thus experience higher shear forces. In contrast, platelets on hydrophobic materials can withstand high shear forces due to strong contact and complete spreading of platelets. When examined with scanning electron microscopy, the platelets on hydrophobic surfaces were indeed more extended like a pancake than on similar material with hydrophilic characteristics [168]. It can be concluded that under conditions of arterial flow, especially at hydrophilic surfaces, less platelets adhere after 15 min due to detachment [169]. It is hypothesised that hydrophilic device surfaces exposed to flowing blood in the human body and under high shear conditions are less likely to accumulate platelets than hydrophobic surfaces.

### 6.2. Roughness

The influence of biomaterial roughness on thrombogenicity is not clear, since various studies show apparently conflicting results. Bailly et al. compared angiographic catheters with different surface roughness by their tendency to become occluded. They concluded that the most thrombogenic material was the smoothest, whereas surface chemistry (polyethylene versus polyamide) contributed to a lesser extent to thrombogenicity [170].

Zingg et al. found that increased roughness caused a decrease in platelet adhesion on hydrophilic surfaces and an increase on hydrophobic surfaces. These results were obtained when flow conditions were applied. During static test conditions no differences between smooth and rough surfaces were found [171, 172]. The higher platelet adhesion on rough hydrophilic cellulose was in agreement with these observations [173].

One explanation for different observations is that thrombus generation is a property of the chemical composition, whereas thrombus adhesion can be related to surface texture [174]. In a more detailed study it was observed that roughness due to titanium crystals appeared to initiate more activation of the clotting cascade, but less platelet adhesion [175]. 

When roughness was increased by a TiO_2_/Ta_2_O_5_ nanofilm on NiTi alloy, the fibrinogen accumulation was enhanced, but platelet adhesion and activation and hemolysis were reduced [176]. This was explained by decreased surface energy, while the roughness was apparently not very important for cell damage. These different effects of two important factors of thrombus formation in conjunction with the variability induced by various flow and shear stress conditions and wettability may explain the conflicting results regarding the thrombogenicity of biomaterials.

**Figure 1:**
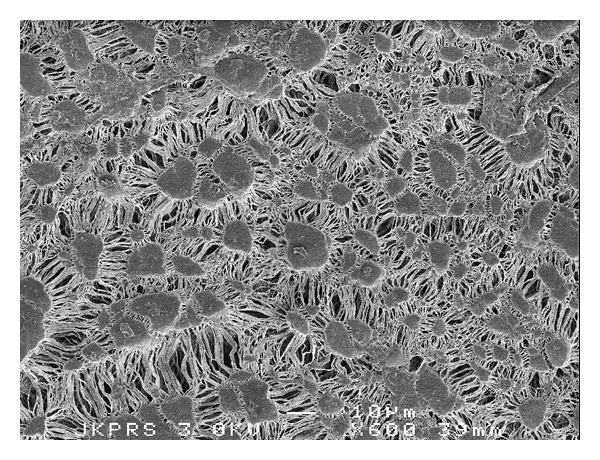
Expanded PTFE provides a rough surface, yet low adhesion of platelets.

The low platelet deposition on expanded PTFE is a well-known example of low adhesion on rough surfaces (Figure 1).

## 7. Reference Materials

**Figure 2:**
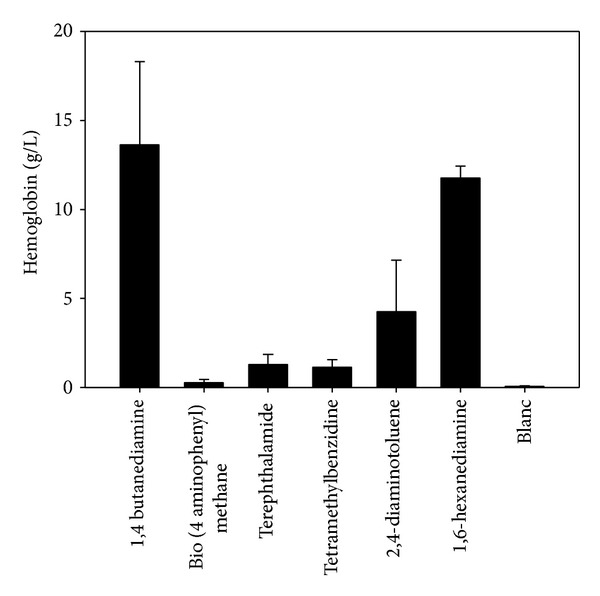
Hemolysis of washed human erythrocytes during 4-hour direct contact with commonly used polymer catalysts.

Reference materials for testing of haemocompatibility are not yet clearly established. ISO 10993/12 only indicates negative references polyvinylchloride and polyurethane. As a consequence the literature shows a variety of positive and negative references, such as silicone rubber and polyethylene [17, 177], glass, nitrocellulose, and expanded polytetrafluorethylene (PTFE) [178], PVC and silicone [179], PTFE, glass, polyamide, and polyethersulfone [180]. The most commonly used reference materials are polyethylene and PVC as negative materials and silicone or latex rubber as positive reference materials. However, all polymers react mildly as compared to most metals. It would be desirable to develop or detect a polymer which is as incompatible as some commonly used metals such as stainless steel or titanium. When only extracts are used, the lubricants, catalysts, or monomers are likely to generate some hemolysis or complement activation. An example of some of the separate polymer components on hemolysis is shown in Figure 2. It is clear that improper polymerisation and subsequent release of polymer monomers may result in severe hemolysis. Slow release will not be detected in 48-hour extracts, although these polymer components may appear clinically relevant [181].

## 8. Acceptance Criteria

A major problem in use of the ISO 10993/4 standard is the lack of acceptance criteria. New types of existing devices can be compared with predicate types and can be accepted when results show similar or better haemocompatibility. However, when no predicate product exists, absolute acceptance criteria should be in place.

The criterium used so far is the percentage hemolysis. 0%–2% is nonhemolytic, 2%–5% is slightly hemolytic, and >5% is hemolytic. The new standard should also include criteria for thrombosis (percentage thrombotic material covering the material surface), platelet count (reduction of platelets below 25% is clinically relevant), coagulation time (using glass as positive reference), and complement activation (to be defined after establishing the optimum incubation time). When possible these criteria should be expressed as percentage of baseline, in which the reference materials serve as high and low quality controls.

## 9. Conclusion

In spite of all the technical improvements made to improve the haemocompatibility of components used for implant devices or external communicating devices, a noticeable activation of plasma proteins and corpuscular blood components still exists. The long-range aim remains the creation of an optimally hemocompatible surface, with which blood would no longer react to humoral and cellular defence mechanisms. Some materials have a poor haemocompatibility but are still used based on their history (silicones) or because of their mechanical properties (metals). A way to improve their haemocompatibility is to apply a coating, such as heparin or polyethylene oxide.

Heparin or NO releasing coatings are merely the beginning of improved haemocompatibility for all materials that come into contact with human blood or tissues. Materials with controlled release of adequate pharmacological substances will be at the surgeon's disposal within the next few years. However, the pharmacologic properties of the implant will be dependent on the purpose and positioning of devices.

Finally, thorough haemocompatibility testing should have a prominent place in the certification of blood contacting medical devices, since a poor haemocompatibility has long-lasting negative effects on the whole body through blood transport of activation products and on functional recovery at the site of implant. Proper methodology and criteria are required to reduce medical devices on the market with poor haemocompatibility.
